# The Relationship Between Gene Subtypes, Symptoms, and Cardiac Function in Patients with Familial Mediterranean Fever

**DOI:** 10.3390/jcm15020862

**Published:** 2026-01-21

**Authors:** Bayram Kızılkaya, Osman Cure, Hüseyin Durak, Mustafa Çetin

**Affiliations:** 1 Department of Internal Medicine, Recep Tayyip Erdoğan University, Training and Research Hospital, Rize 53100, Türkiye; bayram.kizilkaya@saglik.gov.tr; 2 Department of Rheumatology, Faculty of Medicine, Recep Tayyip Erdoğan University, Rize 53100, Türkiye; 3 Department of Cardiology, Faculty of Medicine, Recep Tayyip Erdoğan University, Rize 53100, Türkiyemustafa.cetin@erdogan.edu.tr (M.Ç.)

**Keywords:** familial mediterranean fever, MEFV genotype, phenotype, cardiac function

## Abstract

**Background/Objectives:** Familial Mediterranean fever (FMF) is a chronic autoinflammatory disorder that can affect cardiac structure and function. However, the impact of different Mediterranean fever (MEFV) gene subtypes on clinical features and subclinical cardiac changes remains unclear. This study aimed to evaluate the association between MEFV gene subtypes, clinical features, and cardiac function in patients with FMF. **Methods:** A total of 98 patients with FMF were prospectively included. Twelve mutations in the MEFV gene were screened, and the M694V homozygous (Gene-1), M694V heterozygous (Gene-2), and M680I heterozygous (Gene-3) subtypes were analyzed. All patients underwent transthoracic echocardiography and speckle-tracking strain analysis. **Results:** The age of disease onset was earlier in patients carrying the gene-1 mutation compared to mutation-negative patients (11.4 ± 8.0 and 17.6 ± 11.4 years, respectively; *p* = 0.025). Disease duration was longer in patients with gene-1 mutation (23.3 ± 12.8 and 12.5 ± 9.3 years, respectively; *p* < 0.001), and disease activity score was higher (6.41 ± 1.9 and 5.15 ± 1.6, respectively; *p* = 0.007). Furthermore, left atrial contractile strain was significantly lower in this group (−10.6 ± 3.5% and −14.5 ± 6.1%, respectively; *p* = 0.012). Arthralgia was more frequent in patients with gene-2 mutation (*p* = 0.026), while left atrial contractile strain was better preserved compared to mutation-negative patients (*p* = 0.002). No significant association was found between gene-3 mutation and clinical or cardiac parameters. **Conclusions:** MEFV gene subtypes have different effects on clinical phenotype and cardiac function in FMF. These findings support the importance of genotype-based cardiac monitoring and risk stratification in FMF patients.

## 1. Introduction

Familial Mediterranean Fever (FMF) is a chronic autoinflammatory disease characterized by recurrent episodes of fever and serositis. Mutations in the MEFV gene have been shown to influence disease severity, clinical phenotype, and long-term inflammatory burden. Pyrin dysfunction triggers inflammasome activation, increasing pro-inflammatory cytokines like interleukin-1β, leading to fever, arthritis, and recurrent serositis attacks such as peritonitis, pleuritis, and pericarditis [1,2,3].

FMF is a chronic inflammatory disease with persistent subclinical inflammation and endothelial dysfunction, not limited to attacks [4]. This inflammation can impair the cardiovascular system, causing arterial stiffness, early atherosclerosis, and subclinical myocardial or atrial dysfunction [5]. Cross-sectional studies in adult FMF patients show that even with regular colchicine use and during attack-free periods, right ventricular (RV) global longitudinal strain (GLS) is reduced and myocardial performance, such as the Tei index, is impaired, indicating subclinical RV dysfunction [6]. Genotype and inflammation burden also influence vascular risk, with long-term inflammation causing early vessel wall damage [7].

All these data suggest that FMF is not merely a disease characterized by transient inflammatory attacks; it is a systemic disease with chronic, subclinical inflammation and a risk of cardiovascular remodeling. Although some studies have shown a correlation between certain mutation types, such as MEFV M694V homozygosity, and cardiac findings, these findings are not consistent across all FMF patients. The significant heterogeneity of the phenotype, the preservation of normal cardiac function in some patients, and the small sample sizes of most current studies have made it difficult to clearly establish the genotype–cardiac phenotype relationship [8,9,10]. Although FMF has been associated with subclinical cardiovascular involvement, the effects of specific MEFV genotypes on cardiac structure and function in adult patients remain unclear. Previous studies have largely focused on pediatric populations or used conventional echocardiography without detailed deformation analysis.

To address these limitations, the present study focused on a well-characterized adult FMF cohort and predefined analyses limited to the most common MEFV genotypes with sufficient representation. In addition, standardized clinical assessment and advanced echocardiographic evaluation, including speckle-tracking-derived strain analysis, were performed using a uniform protocol to reduce phenotypic heterogeneity and enhance the detection of genotype-specific subclinical cardiac differences.

Therefore, this study aimed to evaluate the relationship between common MEFV gene subtypes and genotype-specific clinical features and advanced echocardiographic parameters, including speckle-tracking-derived atrial and ventricular strain, in adults with FMF. We hypothesized that FMF patients carrying different MEFV genotypes would exhibit distinct patterns of subclinical cardiac involvement, particularly with regard to left atrial strain parameters.

## 2. Methods

### 2.1. Study Design

A total of 98 individuals meeting the Tel Hashomer diagnostic criteria for FMF [11] were prospectively enrolled in this consultation-based cohort study. This study was approved by the Ethics Committee of Recep Tayyip Erdoğan University Faculty of Medicine (Date: 9 October 2025, No: 2025/425). All procedures were carried out in accordance with the ethical principles outlined in the Declaration of Helsinki. Informed consent was obtained from all participants.

### 2.2. Participants and Clinical and Laboratory Evaluation

Inclusion criteria were FMF patients diagnosed by Tel Hashomer criteria, able to undergo genetic testing and echocardiography, and on long-term colchicine. Exclusion criteria included cardiac diseases (e.g., congenital heart disease, valvular disease, heart failure), malignant, renal, or hepatic disease, active/chronic infections or inflammation, and echocardiography technical limitations. FMF diagnosis requires ≥1 major or ≥2 minor Tel Hashomer criteria: majör recurrent fever with serositis (peritonitis, pleuritis, synovitis), AA amyloidosis without underlying disorder, or clear colchicine response; minor recurrent febrile episodes, erysipelas-like rash, or positive family history [11].

A detailed questionnaire recorded demographics, attack-related symptoms (fever, abdominal pain, diarrhea/constipation, arthritis/joint/muscle pain, pleuritic/chest pain, erysipelas-like erythema, family history), disease duration, activity score, and medication use. After ≥8 h fasting, venous blood was drawn for labs including complete blood count, fasting glucose, kidney function, total protein, albumin, and C-reactive protein (CRP). Diabetes mellitus (DM) was defined as fasting plasma glucose ≥ 126 mg/dL, hemoglobin A1c (HbA1c) ≥ 6.5%, or use of antidiabetic medication. Hyperlipidemia is defined as the presence of low-density lipoprotein (LDL) cholesterol ≥ 130 mg/dL, total cholesterol ≥ 200 mg/dL, triglycerides ≥ 150 mg/dL, or high-density lipoprotein (HDL) cholesterol < 40 mg/dL in men and <50 mg/dL in women in the blood. These comorbidities were present before FMF diagnosis [12,13].

### 2.3. Genetic Analysis of MEFV Mutations in FMF Patients

Genomic DNA was extracted from peripheral blood using the Blood Kit (SNP Biotechnology R&D Ltd., Ankara, Türkiye). Twelve common MEFV mutations (E148Q, P369S, F479L, M680I [G/A, G/C], I692del, M694V, M694I, K695R, V726A, A744S, R761H) were analyzed using a StripAssay-based method (SNP Biotechnology R&D Ltd., Ankara, Türkiye), which amplifies target regions by polymerase chain reaction (PCR, hybridizes them to mutation-specific probes, and detects signals via enzymatic color development. The M694V homozygous (Gene-1), M694V heterozygous (Gene-2), and M680I heterozygous (Gene-3) subtypes—common in FMF and linked to pronounced clinical phenotypes—were examined; rare genotypes were excluded due to low numbers.

### 2.4. Echocardiographic Assessment

Transthoracic echocardiography was performed in all participants using a Vivid E95 ultrasound system (Ultra Edition; GE Healthcare, Horten, Norway) equipped with an M5Sc transducer. All examinations were carried out by a single cardiologist with more than five years of experience in echocardiographic imaging, who was blinded to genotype status. All measurements were recorded by averaging three consecutive heart cycles. Image acquisition, measurement standards, and deformation analysis followed the recommendations of the American Society of Echocardiography.

Conventional echocardiographic assessment included left ventricular chamber dimensions, interventricular septal and posterior wall thickness, and calculation of left ventricular ejection fraction (LVEF) using the modified Simpson method. The left ventricular mass was derived using the Devereux-modified ASE formula, and the left ventricular mass index (LV mass index) was derived by indexing LV mass to body surface area.

Diastolic function parameters included mitral inflow velocities (E and A waves), E/A ratio, lateral tissue Doppler velocities (e′, a′, and s′), and deceleration time. Doppler flow assessment and evaluation for presystolic wave (PSW) were performed at the proximal left ventricular output track (LVOT) in the apical five-chamber (A5C) view. Left atrial volume index (LAVI) was measured using the biplane area–length method from apical two- and four-chamber views at end-systole and indexed to body surface area.

Right heart parameters included tricuspid annular plane systolic excursion (TAPSE), right ventricular fractional area change (RV FAC), tricuspid lateral s′ velocity, and right atrial volume.

Speckle-tracking echocardiography was performed for myocardial deformation analysis. Left ventricular global longitudinal strain (LVGLS) was calculated by averaging strain values obtained from 18 segments across the apical four-chamber (A4C), two-chamber (A2C), and three-chamber (A3C) views. Left atrial strain was quantified using dedicated deformation software (EchoPAC version 204, revision 73, GE Healthcare) and categorized into reservoir (LA Strain 1), conduit (LA Strain 2), and contractile strain (LA Strain 3), corresponding, respectively, to ventricular systole, early diastole, and atrial contraction. QRS onset was used as the time reference [14].

All echocardiographic images were digitally stored and analyzed offline to ensure consistency and reproducibility of the measurements.

### 2.5. Statistical Analyses

All statistical analyses were performed using IBM SPSS Statistics software (version 25.0; IBM Corp., Armonk, NY, USA). Continuous variables were examined for normality using the Shapiro–Wilk test. Normally distributed continuous variables were presented as mean ± standard deviation (SD) and compared between mutation-positive and mutation-negative groups using the independent samples *t*-test. Non-normally distributed variables were expressed as median (interquartile range) and compared using the Mann–Whitney U test.

Categorical variables were summarized as counts and percentages, and group comparisons were performed using the chi-square test or Fisher’s exact test when appropriate. Genotype–phenotype comparisons were conducted separately for each MEFV locus Gene-1 (M694V homozygous), Gene-2 (M694V heterozygous), and Gene-3 (M680I heterozygous) All echocardiographic parameters including chamber dimensions, functional indices, and strain measurements were analyzed using the same distribution-based statistical approach.

No missing data imputation was required, as complete datasets were available for all clinical and echocardiographic variables. Given the exploratory nature of the study and the relatively small number of mutation-positive patients in some subgroups, no corrections were applied for multiple comparisons. A two-tailed *p* value < 0.05 was considered statistically significant.

## 3. Results

### 3.1. Study Population and Demographics

**Figure 1:**
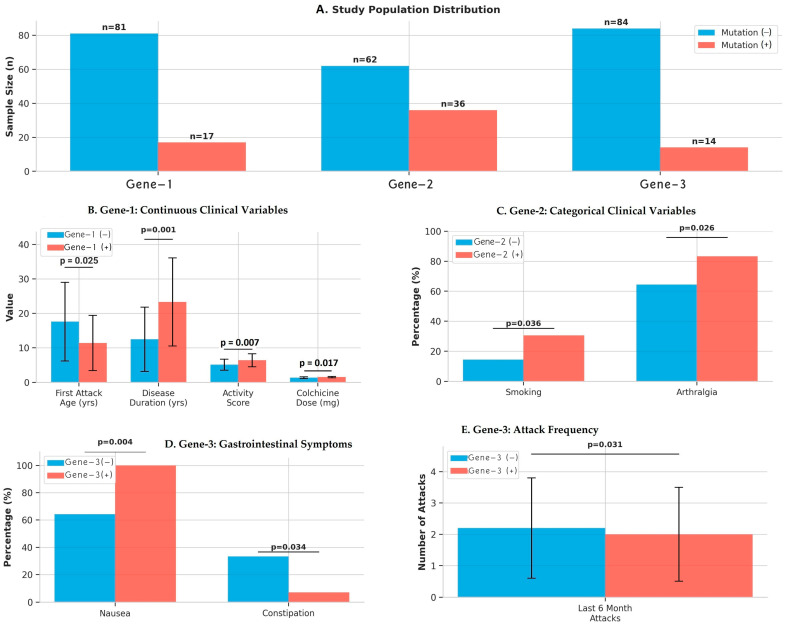
Clinical Characteristics of FMF Patients According to MEFV Genotype.

The study cohort comprised 98 patients with clinically diagnosed FMF who underwent comprehensive clinical evaluation and echocardiographic assessment. Although 12 MEFV mutations were screened, only variants at the Gene-1, Gene-2, and Gene-3 loci were observed with sufficient frequency to enable statistically robust subgroup analyses and were therefore retained for the final analysis. Genetic analysis identified MEFV gene mutations across three specific loci: Gene-1, Gene-2, and Gene-3. The comparison of baseline, demographic, laboratory and clinical data of mutation-positive and -negative groups in Gene-1, Gene-2 and Gene-3 analysis is shown in detail in Table 1 and Table 2. For Gene-1, 17 patients (17.3%) were mutation-positive while 81 patients (82.7%) were mutation-negative. Gene-2 mutations were identified in 36 patients (36.7%), with 62 patients (63.3%) being mutation-negative. Gene-3 mutations were the least prevalent, detected in 14 patients (14.3%), while 84 patients (85.7%) were mutation-negative. All patients underwent standardized clinical assessments including detailed symptom history, disease activity scoring, and medication documentation. Baseline demographics, laboratory parameters, and comprehensive echocardiographic evaluations were obtained for all participants. The distribution of mutation-positive and mutation-negative patients across the three genotypes allowed for robust statistical comparisons of clinical and cardiac phenotypes. Figure 1 summarizes clinical characteristics by MEFV genotype, illustrating significant group differences in continuous variables, attack frequency, categorical clinical features, and symptom profiles across Gene-1, Gene-2, and Gene-3 subgroups.

### 3.2. Clinical Characteristics by Genotype

#### 3.2.1. Gen-1 Mutation (M694V Homozygous)

Patients carrying the gene-1 mutation had an earlier onset of disease (11.4 ± 8.0 vs. 17.6 ± 11.4 years, ***p* = 0.025**), a longer duration of disease (23.3 ± 12.8 vs. 12.5 ± 9.3 years, ***p* < 0.001**), and a higher disease activity score (6.41 ± 1.9 vs. 5.15 ± 1.6, ***p* = 0.007**). These patients also used higher doses of colchicine (*p* = 0.017). No significant differences were found between the groups in terms of demographic characteristics, comorbidities, and laboratory parameters (Table 1 and Table 2, and Figure 1).

#### 3.2.2. Gene-2 Mutation (M694V Heterozygous)

Patients with the gene-2 mutation had a higher prevalence of smoking (30.6% vs. 14.5%, ***p* = 0.036**) and arthralgia (83.3% vs. 64.5%, ***p* = 0.026**). No significant differences were observed between the groups in terms of age of onset, duration of disease, activity score, classic FMF symptoms, laboratory findings, and colchicine dose (Table 1 and Table 2 and Figure 1).

#### 3.2.3. Gene-3 Mutation (M680I Heterozygous)

Patients with the gene-3 mutation had a lower number of attacks in the last 6 months (***p* = 0.031**). While nausea was observed in all patients in this group (100% vs. 64.3%, ***p* = 0.004**), constipation was less frequent (7.1% vs. 33.3%, *p* = 0.034). No significant differences were found in other clinical features, disease activity, and treatment requirements (Table 1 and Table 2 and Figure 1).

### 3.3. Echocardiographic Findings by Genotype

**Figure 2:**
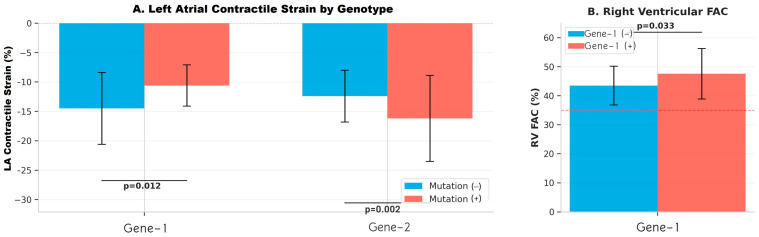
Echocardiographic Parameters According to MEFV Genotype in FMF Patients.

**Figure 3:**
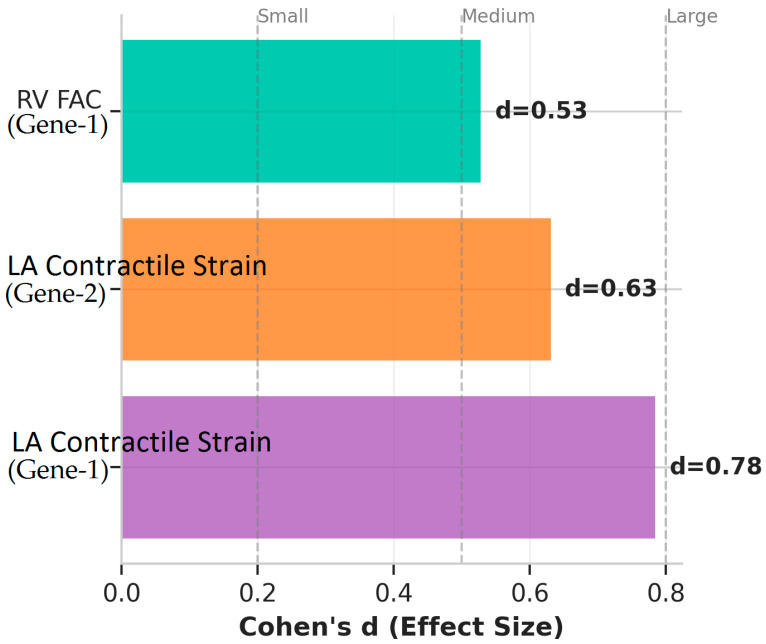
Cohen’s d Effect Sizes for Echocardiographic Parameters by MEFV Genotype.

The comparison of echocardiographic data of mutation-positive and -negative groups in Gen-1, Gene-2 and Gene-3 analysis is shown in detail in Table 3. Figure 2 and Figure 3 present genotype-specific differences in echocardiographic parameters, highlighting significant reductions in LA contractile strain in Gene-1 and Gene-2 mutation-positive patients, a higher right ventricular fractional area change (RV FAC) in Gene-1, and corresponding effect sizes supporting these findings.

#### 3.3.1. Gene-1 Mutation and Cardiac Parameters

Comprehensive echocardiographic assessment revealed significant differences in cardiac function parameters between Gene-1 mutation groups. Left atrial strain analysis demonstrated that Gene-1 mutation-positive patients had reduced LA contractile strain values (−10.6 ± 3.5% vs. −14.5 ± 6.1%, ***p* = 0.012**), indicating impaired left atrial contractile function. Less negative strain values reflect diminished atrial deformation and suggest early atrial myopathy in mutation carriers. Interestingly, right ventricular function assessment showed higher RV FAC in mutation-positive patients (47.6 ± 8.7% vs. 43.5 ± 6.7%, ***p* = 0.033**), suggesting preserved or potentially enhanced right ventricular systolic function. Both groups maintained RV FAC values within normal range (>35%), though the difference between groups was statistically significant. Left ventricular parameters including ejection fraction, LV global longitudinal strain (LVGLS), and mass index showed no significant differences between Gene-1 mutation groups. Diastolic function parameters including mitral inflow velocities (E/A ratio), tissue Doppler measurements (E/E′ ratio), and deceleration time were comparable between groups. Left atrial volume index, tricuspid annular plane systolic excursion (TAPSE), and right atrial volume showed no significant differences.

#### 3.3.2. Gene-2 Mutation and Cardiac Parameters

Gene-2 mutation analysis revealed a contrasting pattern of atrial function compared to Gene-1 (Figure 2). Notably, Gene-2 mutation-negative patients demonstrated lower LA contractile strain values (−12.4 ± 4.4% vs. −16.2 ± 7.3%, ***p* = 0.002**), indicating that mutation-positive patients had better preserved LA contractile function. This finding represents an inverse relationship compared to Gene-1, where mutation-positive status was associated with worse atrial contractile function. The highly significant *p*-value (***p* = 0.002**) underscores the robustness of this genotype-specific difference. LVGLS showed a trend toward better values in Gene-2 mutation-positive patients (***p* = 0.071**), though this did not reach statistical significance. This trend suggests potential preservation of left ventricular systolic function in Gene-2 mutation carriers, consistent with the atrial findings. Standard echocardiographic parameters including left ventricular ejection fraction, chamber dimensions, and wall thickness measurements were similar between Gene-2 mutation groups. Diastolic function parameters, right ventricular function indices, and valvular assessments showed no significant differences.

#### 3.3.3. Gene-3 Mutation and Cardiac Parameters

Echocardiographic analysis of Gene-3 mutation groups revealed limited significant differences, with one notable trend in diastolic function (Figure 2). Deceleration time (DT) showed a trend toward shorter values in mutation-positive patients (138 ± 77 ms vs. 164 ± 47 ms, *p* = 0.087), though this did not achieve statistical significance. Shorter deceleration time may suggest altered left ventricular relaxation patterns or increased left atrial pressure, potentially indicating subclinical diastolic dysfunction in Gene-3 mutation carriers. The wide standard deviation in the mutation-positive group (±77 ms) suggests heterogeneity in diastolic function within this genotype. Left ventricular systolic function parameters including ejection fraction and LVGLS were comparable between groups. Left atrial strain measurements, right ventricular function indices, and chamber dimensions showed no significant differences between Gene-3 mutation-positive and mutation-negative patients. Additional echocardiographic parameters including mitral inflow patterns, tissue Doppler velocities, and valvular assessments were similar across mutation groups. Figure 1 and Figure 2 present additional visualizations of echocardiographic data across all genotypes and illustrate the relationships between disease severity markers and cardiac function parameters.

## 4. Discussion

This study evaluated the effects of MEFV gene subtypes on clinical phenotype and cardiac function in FMF patients. The findings showed that different mutations can have different effects not only on disease severity and symptom profile, but also on subclinical cardiac function. In particular, patients carrying the homozygous M694V mutation showed a more severe clinical picture and decreased left atrial contractile strain and subclinical atrial dysfunction, while cardiac effects were more heterogeneous or milder in other genotypes.

Looking at the literature, it has been reported that the homozygous M694V genotype is associated with amyloidosis and a heavier inflammatory burden in FMF patients. The M694V exon 10 mutation has been associated with a more severe disease phenotype and an earlier onset risk. It has also been reported that the homozygous M694V genotype is associated with more severe disease and colchicine resistance [15,16,17]. Similarly to the literature, in our study, patients carrying the Gene-1 (M694V homozygous) mutation showed a more severe clinical phenotype with higher disease activity scores, earlier age of onset, and longer disease duration.

The decrease in left atrial (LA) contractile strain values as an indicator of subclinical atrial dysfunction in FMF patients suggests that chronic inflammation may have a negative effect on atrial mechanics; similarly, previous studies have shown that cardiac function assessments are important in FMF. Studies using speckle-tracking echocardiography have reported changes such as a decrease in right ventricular global longitudinal strain values and an increase in myocardial performance index in FMF patients, which indicates subclinical right ventricular dysfunction [6,18]. In our study, the contrast atrial function pattern in the Gene-2 heterozygous mutation group shows that the cardiac effects of different MEFV genotypes are not homogeneous. Genotype-specific cardiac differences in FMF likely reflect the cumulative effects of chronic inflammation and disease burden. In our cohort, M694V homozygous patients, with earlier onset and longer disease duration, exhibited subclinical left atrial dysfunction, possibly due to persistent systemic inflammation and myocardial stress. In contrast, M694V heterozygotes showed relatively preserved atrial strain, suggesting a lower inflammatory burden or compensatory cardiac adaptations. Subtle diastolic changes observed in M680I carriers may indicate early or mild myocardial involvement. These observations highlight the complex interplay between genotype, inflammation, and cardiac remodeling, emphasizing the importance of genotype-informed cardiovascular monitoring in adult FMF patients.

The diastolic function trend observed in patients carrying the Gene-3 (M680I heterozygous) mutation suggests that cardiac effects in FMF may vary depending on the mutation type and that some genotypes may present with more insidious subclinical findings. This reflects the complexity of the effects of inflammation on the cardiovascular system and parallels the findings of other studies that associate subclinical vascular damage indicators such as arterial stiffness and atherosclerotic changes [19,20].

Studies in FMF patients have shown impaired coronary microvascular function and left ventricular diastolic function, and inflammatory markers such as high hs-CRP levels have been found to correlate with these findings; the effect of inflammation on the cardiovascular system is supported by these findings [21,22]. In addition, some genotype–phenotype relationships have shown that rare mutation types can also be associated with cardiac findings; for example, there are findings such as the association of the E148Q/V726A mutation with pericardial effusion in children [8]. However, such relationships have not been consistently demonstrated in the adult population in general, which shows that the effect of genetic heterogeneity on clinical outcomes in FMF is more complex.

Extensive reviews report that FMF is associated with increased cardiovascular risk and subclinical vascular disease, likely due to chronic inflammation affecting the endothelium and atherosclerosis [7,23,24]. These mechanisms align with our findings and may explain why different MEFV genotypes impact cardiac function differently. This study is among few adult cohort studies assessing the FMF genotype–cardiac phenotype relationship. Unlike most pediatric or small-sample studies, our results suggest that genotypes may differently affect cardiac function, highlighting the need for larger studies to clarify genotype-dependent inflammatory effects on the cardiovascular system.

## 5. Limitations

This study has several limitations. It was conducted at a single center in a specific population; therefore, genotype–phenotype relationships may vary across different ethnic and geographic groups. Rare MEFV mutations were excluded due to insufficient representation, which may introduce bias and limit the generalizability of genotype–phenotype associations. Restricting analyses to the most prevalent genotypes allowed for more robust and internally valid comparisons. In addition, multiple comparisons were performed across clinical and echocardiographic parameters, increasing the risk of type I error; therefore, findings should be interpreted with caution. The cross-sectional design of the study precludes causal inference, and the absence of longitudinal cardiovascular follow-up limits the assessment of long-term outcomes. The lack of advanced cardiac imaging modalities, such as cardiac magnetic resonance imaging, may have limited the detection of subtle myocardial changes. Larger multicenter studies including rarer mutations and longitudinal follow-up are needed to confirm and extend these findings.

## 6. Conclusions

This study shows that MEFV gene subtypes influence clinical and cardiac phenotypes in FMF. The M694V homozygous mutation was linked to more severe symptoms and subclinical atrial dysfunction, while M694V heterozygous patients had a contrasting cardiac profile, and M680I heterozygous carriers showed milder effects. These findings suggest that genotype-based cardiac monitoring could personalize risk assessment. Larger prospective studies are needed to clarify genotype effects on cardiac morbidity and long-term outcomes.

## Figures and Tables

**Table 1:** Demographic and Clinical Characteristics of the Study Population According to Genotype Groups (Gene-1, Gene-2, Gene-3).

Variable	Gene-1 Mutation (–) *n* = 81	Gene-1 Mutation (+) *n* = 17	*p*	Gene-2 Mutation (–) *n* = 62	Gene-2 Mutation (+) *n* = 36	*p*	Gene-3 Mutation (–) *n* = 84	Gene-3 Mutation (+) *n* = 14	*p*
Age (years)	34.8 ± 12.1	39.0 ± 14.2	NS	36.4 ± 13.2	33.9 ± 11.3	NS	34.8 ± 12.1	39.0 ± 14.2	NS
Sex (female, %)	37 (45.7%)	8 (47.1%)	NS	27 (43.5%)	18 (50%)	NS	41 (48.8%)	4 (28.6%)	NS
Smoking (%)	17 (21%)	3 (17.6%)	NS	9 (14.5%)	11 (30.6%)	0.036	18 (21.4%)	2 (14.3%)	NS
BMI (kg/m^2^)	26.3 ± 4.8	26.2 ± 5.2	NS	26.5 ± 5.2	26.1 ± 4.4	NS	26.3 ± 7.8	26.5 ± 6.1	NS
Diabetes mellitus (%)	8 (9.9%)	1 (5.9%)	NS	4 (6.5%)	5 (13.9%)	NS	8 (9.5%)	1 (7.1%)	NS
Hypertension (%)	4 (4.9%)	3 (17.6%)	0.082	6 (9.7%)	1 (2.8%)	NS	7 (8.3%)	0 (0%)	NS
Hyperlipidemia (%)	32 (39.5%)	7 (41.2%)	NS	27 (43.5%)	12 (33.3%)	NS	32 (38.1%)	7 (50%)	NS
Age at first attack (years)	17.6 ± 11.4	11.4 ± 8.0	0.025	16.4 ± 10.3	16.6 ± 12.7	NS	17.6 ± 11.4	11.0 ± 8.0	NS
Age at diagnosis (years)	25.8 ± 11.5	24.6 ± 17.5	NS	25.5 ± 13.2	25.6 ± 11.8	NS	25.8 ± 11.5	24.6 ± 17.0	0.099
Disease duration (years)	12.5 ± 9.3	23.3 ± 12.8	<0.001	15.8 ± 12.3	11.8 ± 6.6	0.074	12.5 ± 9.3	23.2 ± 12.8	—
FMF severity score	1.98 ± 0.35	2.10 ± 0.40	NS	2.00 ± 0.40	1.97 ± 0.30	NS	1.98 ± 0.30	2.06 ± 0.40	NS
Activity score	5.15 ± 1.6	6.41 ± 1.9	0.007	5.4 ± 1.8	5.3 ± 1.6	NS	5.1 ± 1.6	6.4 ± 1.9	NS
Attacks in last 6 months	2.27 ± 1.6	2.0 ± 1.6	NS	2.1 ± 1.5	2.4 ± 1.6	NS	2.2 ± 1.6	2.0 ± 1.5	0.031
Family history of FMF (%)	44 (54.3%)	12 (70.6%)	NS	37 (59.7%)	19 (52.8%)	NS	47 (56%)	9 (64.3%)	NS
Fever (%)	64 (79%)	14 (82.4%)	NS	49 (79%)	29 (80%)	NS	66 (78.6%)	12 (85.7%)	NS
Abdominal pain (%)	80 (98.8%)	17 (100%)	NS	61 (98.4%)	36 (100%)	NS	83 (98.8%)	14 (100%)	NS
Pleuritic pain (%)	39 (48.1%)	7 (41.2%)	NS	30 (48.4%)	16 (44.4%)	NS	42 (50%)	4 (28.6%)	0.079
Chest pain (%)	48 (59.3%)	11 (64.7%)	NS	39 (62.9%)	20 (55.6%)	NS	53 (63.1%)	6 (42.9%)	0.089
Arthritis (%)	9 (11.1%)	1 (5.9%)	NS	6 (9.7%)	4 (11.1%)	NS	7 (8.3%)	3 (21.4%)	NS
Arthralgia (%)	57 (70.4%)	13 (76.5%)	NS	40 (64.5%)	30 (83.3%)	0.026	59 (70.2%)	11 (78.6%)	NS
Rash (%)	8 (9.9%)	1 (5.9%)	NS	5 (8.1%)	4 (11.1%)	NS	7 (8.3%)	2 (14.3%)	NS
Diarrhea (%)	21 (25.9%)	5 (29.4%)	NS	17 (27.4%)	9 (25%)	NS	22 (26.2%)	4 (28.6%)	NS
Nausea (%)	55 (67.9%)	13 (76.5%)	NS	46 (74.2%)	22 (61.1%)	0.072	54 (64.3%)	14 (100%)	0.004
Myalgia (%)	58 (71.6%)	13 (76.5%)	NS	45 (72.6%)	26 (72.2%)	NS	61 (72.6%)	10 (71.4%)	NS
Constipation (%)	24 (29.6%)	5 (29.4%)	NS	18 (29%)	11 (30.6%)	NS	28 (33.3%)	1 (7.1%)	0.034
ACE/ARB use (%)	3 (3.7%)	2 (11.8%)	NS	4 (6.5%)	1 (2.8%)	NS	5 (6%)	0 (0%)	NS
CCB use (%)	3 (3.7%)	2 (11.8%)	NS	5 (8.1%)	0 (0%)	NS	5 (6%)	0 (0%)	NS
Colchicine dose (mg/day)	1.35 ± 0.28	1.53 ± 0.22	0.017	1.4 ± 0.3	1.36 ± 0.25	NS	1.35 ± 0.3	1.53 ± 0.22	NS

Continuous variables are presented as mean ± standard deviation (SD). Categorical variables are presented as number and percentage (%). NS: Not Significant. FMF: Familial Mediterranean Fever, BMI: Body Mass Index ACE: Angiotensin Converting Enzyme, ARB: Angiotensin Receptor Blocker; CCB: Calcium Channel Blocker.

**Table 2:** Laboratory Parameters of the Study Population According to Genotype Groups (Gene-1, Gene-2, Gene-3).

Variable	Gene-1 Mutation (–) *n* = 81	Gene-1 Mutation (+) *n* = 17	*p*	Gene-2 Mutation (–) *n* = 62	Gene-2 Mutation (+) *n* = 36	*p*	Gene-3 Mutation (–) *n* = 84	Gene-3 Mutation (+) *n* = 14	*p*
WBC (×10^3^/µL)	7.3 ± 2.0	7.2 ± 2.1	NS	7.2 ± 1.9	7.6 ± 2.1	NS	7.3 ± 2.1	7.2 ± 2.1	NS
Neutrophils (×10^3^/µL)	4.6 ± 2.1	4.6 ± 1.8	NS	4.5 ± 1.7	4.8 ± 2.3	NS	4.6 ± 2.1	4.6 ± 1.8	NS
Lymphocytes (×10^3^/µL)	2.1 ± 0.5	1.9 ± 0.4	NS	2.03 ± 0.5	2.08 ± 0.6	NS	2.1 ± 0.6	1.9 ± 0.5	NS
Hemoglobin (g/dL)	13.5 ± 1.8	12.8 ± 1.5	NS	13.2 ± 1.7	13.7 ± 1.8	NS	13.5 ± 1.8	12.9 ± 1.4	NS
Total protein (g/dL)	7.5 ± 0.5	7.5 ± 0.4	NS	7.5 ± 0.4	7.6 ± 0.4	NS	7.5 ± 0.5	7.5 ± 0.4	NS
Albumin (g/dL)	4.5 ± 0.5	4.4 ± 0.3	NS	4.5 ± 0.5	4.5 ± 0.4	NS	4.5 ± 0.5	4.4 ± 0.3	NS
Fibrinogen (mg/dL)	341.8 ± 110	336 ± 104	NS	336 ± 99	348 ± 125	NS	341 ± 110	336 ± 104	NS
Serum creatinine (mg/dL)	0.87 ± 0.07	0.91 ± 0.08	NS	0.90 ± 0.20	0.75 ± 0.17	NS	0.87 ± 0.07	0.90 ± 0.81	NS
eGFR (mL/min/1.73 m^2^)	107.1 ± 23.3	105.3 ± 30.2	NS	105.7 ± 27.8	108.6 ± 17.0	NS	107 ± 23	105.3 ± 30	NS
Glucose (mg/dL)	90.7 ± 9.5	87.3 ± 7.8	NS	88.7 ± 8.6	92.5 ± 10.1	0.060	90.7 ± 9.5	87.2 ± 7.9	NS
ESR (mm/h)	9.6 ± 8.4	13.6 ± 7.5	0.076	10.8 ± 8.1	9.4 ± 8.9	NS	9.6 ± 8.4	13.6 ± 7.4	NS
CRP (mg/L)	5 (2–8.5)	8.2 (2.4–13.5)	NS	5 (2–10)	5.4 (2.3–9.7)	NS	5.5 (2.3–10)	3.2 (1.1–9)	NS

Continuous variables are presented as mean ± standard deviation (SD). Categorical variables are presented as number and percentage (%). NS: Not Significant. WBC: White Blood Count; eGFR: Estimated Glomerular filtration rate; ESR: Erythrocyte Sedimentation Rate; CRP: C-Reactive Protein.

**Table 3:** Echocardiographic Characteristics According to Genotype Groups (Gene-1, Gene-2, Gene-3).

Variable	Gene-1 Mutation (–) *n* = 81	Gene-1 Mutation (+) *n* = 17	*p*	Gene-2 Mutation (–) *n* = 62	Gene-2 Mutation (+) *n* = 36	*p*	Gene-3 Mutation (–) *n* = 84	Gene-3 Mutation (+) *n* = 14	*p*
LVEF (%)	60.2 ± 5.5	60.1 ± 5.8	NS	60.0 ± 5.1	60.5 ± 6.4	NS	60.0 ± 5.8	61.4 ± 3.9	NS
LV Mass Index (g/m^2^)	77.1 ± 21.9	77.3 ± 13.9	NS	77.1 ± 22.8	77.0 ± 16.6	NS	78.1 ± 19.9	70.8 ± 24.6	NS
LVGLS (%)	−19.6 ± 1.3	−19.1 ± 1.4	NS	−19.3 ± 1.1	−19.8 ± 1.6	0.071	−19.6 ± 1.4	−18.9 ± 0.8	NS
LAVI (mL/m^2^)	18.2 ± 4.8	19.2 ± 2.7	NS	18.7 ± 4.6	17.8 ± 4.4	NS	18.2 ± 4.4	19.2 ± 5.1	NS
LA Reservoir Strain (%)	−34.8 ± 11.2	−31.2 ± 5.9	NS	−34.3 ± 7.8	−34.3 ± 14.1	NS	−34.3 ± 11.0	−34.3 ± 6.6	NS
LA conduit Strain (%)	−19.9 ± 6.1	−18.7 ± 6.5	NS	−19.7 ± 6.8	−19.5 ± 5.8	NS	−19.7 ± 6.4	−19.0 ± 4.6	NS
LA contractile Strain (%)	−14.5 ± 6.1	−10.6 ± 3.5	**0.012**	−12.4 ± 4.4	−16.2 ± 7.3	**0.002**	−13.9 ± 6.2	−13.3 ± 3.6	NS
Mitral E (cm/s)	82.2 ± 20.3	76.8 ± 22.5	NS	81.5 ± 22.6	80.8 ± 16.8	NS	80.2 ± 17.9	85.6 ± 33.0	NS
Mitral A (cm/s)	65.3 ± 15.6	59.1 ± 21.1	NS	63.1 ± 17.6	66.2 ± 15.5	NS	64.9 ± 17.5	60.3 ± 11.4	NS
Lateral e′ (cm/s)	14.7 ± 3.2	14.0 ± 3.4	NS	14.3 ± 3.2	15.1 ± 3.2	NS	14.7 ± 3.0	13.9 ± 4.2	NS
Lateral a′ (cm/s)	9.5 ± 2.5	8.7 ± 2.1	NS	9.4 ± 2.5	9.4 ± 2.4	NS	9.5 ± 2.5	9.0 ± 2.5	NS
Lateral s′ (cm/s)	10.3 ± 2.9	9.2 ± 1.7	NS	10.0 ± 2.6	10.3 ± 3.1	NS	10.0 ± 2.7	10.7 ± 3.2	NS
E/A ratio	1.3 ± 0.29	1.3 ± 0.4	NS	1.3 ± 0.4	1.26 ± 0.3	NS	1.3 ± 0.3	1.4 ± 0.5	NS
E/e′ ratio	5.8 ± 1.8	5.8 ± 2.5	NS	5.9 ± 2.1	5.5 ± 1.5	NS	5.6 ± 1.7	6.6 ± 2.9	NS
Deceleration time (ms)	159 ± 52	166 ± 58	NS	160 ± 59	161 ± 40	NS	164 ± 47	138 ± 77	0.087
TAI	0.53 ± 0.13	0.52 ± 0.21	NS	0.52 ± 0.15	0.54 ± 0.14	NS	0.53 ± 0.15	0.53 ± 0.10	NS
TAPSE (mm)	22.1 ± 4.3	22.7 ± 4.2	NS	22.1 ± 3.1	22.3 ± 2.5	NS	22.1 ± 3.0	22.2 ± 2.0	NS
Aortic diameter (mm)	28.6 ± 2.9	28.1 ± 3.5	NS	28.2 ± 3.2	29.0 ± 2.6	NS	28.7 ± 3.0	27.5 ± 3.0	NS
RV FAC (%)	43.5 ± 6.7	47.6 ± 8.7	**0.033**	45.1 ± 7.3	42.9 ± 7.1	NS	44.6 ± 7.2	41.8 ± 7.1	NS
RA Volume (mL)	29.1 ± 9.1	26.2 ± 6.5	NS	28.8 ± 8.9	28.1 ± 8.7	NS	28.2 ± 8.6	30.7 ± 10.0	NS
PSW (%)	22 (27.2%)	5 (29.4%)	NS	18 (29%)	9 (25%)	NS	23 (27.4%)	4 (28.6%)	NS

Continuous data are shown as mean ± standard deviation (SD). Categorical variables are presented as numbers and percentages. Bold *p*-values indicate statistical significance (<0.05). LVEF = Left ventricular ejection fraction; LVGLS: LV global longitudinal strain; LAVI: LA volume index; FAC: Fractional area change; TAPSE: Tricuspid annular plane systolic excursion; TAI: Tei index; RV FAC: Right Ventricular Fractional Area Change; RA Volume: Right Atrial Volume; PSW: Post-Systolic Wall motion.

## Data Availability

The original contributions presented in this study are included in the article. Further inquiries can be directed to the corresponding author.
